# Supplementary material for: Intracerebral Administration of a Ligand-ASO Conjugate Selectively Reduces α-Synuclein Accumulation in Monoamine Neurons of Double Mutant Human A30P*A53T*α-Synuclein Transgenic Mice
Source: Int J Mol Sci. 2021 Mar 13;22(6):2939. doi: 10.3390/ijms22062939 (PMC8001805; doi:10.3390/ijms22062939)
Supplement: Supplementary file 1 [file ijms-22-02939-s001.pdf]

# *Supplementary Materials*

# Intracerebral Administration of a Ligand-ASO Conjugate Selectively Reduces *α*-Synuclein Accumulation in Monoamine Neurons of Double Mutant Human A30P*A53T**α*-Synuclein Transgenic Mice

**Rubén Pavia-Collado [1,2,3], Valentín Cóppola-Segovia [4], Lluís Miquel-Rio [1,2,3], Diana Alarcón-Aris [1,2], Raquel Rodríguez-Aller [5], María Torres-López [1,2], Verónica Paz [1,2,3], Esther Ruiz-Bronchal [1,2,3], Leticia Campa [1,2,3], Francesc Artigas [1,2,3], Andrés Montefeltro [6], Raquel Revilla [6] and Analia Bortolozzi [1,2,3,*]**

1. Institut d'Investigacions Biomèdiques de Barcelona (IIBB), Spanish National Research Council (CSIC), Barcelona, Spain; ruben.pavia@iibb.csic.es (R.P.-C.); lluis.miquel@iibb.csic.es (L.M.-R.); d.alarcon@keval.es (D.A.-A.); maria.torres@iibb.csic.es (M.T.-L.); veronica.paz@iibb.csic.es (V.P.); esther.ruiz@iibb.csic.es (E.R.-B.); lcmnqi@iibb.csic.es (L.C.); fapnqi@iibb.csic.es (F.A.)
2. Institut d'Investigacions Biomèdiques August Pi i Sunyer (IDIBAPS), Barcelona, Spain
3. Centro de Investigación Biomédica en Red de Salud Mental (CIBERSAM), ISCIII, Madrid, Spain
4. Laboratory of Neurobiology and Redox Pathology, Department of Basic Pathology, Federal University of Paraná (UFPR), Curitiba, Brazil; valen.coppola@gmail.com
5. CHU de Quebec Research Center, Axe Neurosciences. Department of Molecular Medicine, Faculty of Medicine, Université Laval, CERVO Brain Research Centre, Quebec City, QC, Canada; raquel.rodriguez-aller.1@ulaval.ca
6. n-Life Therapeutics, S.L., Granada, Spain; amontefeltro@lingeamc.com (A.M.); rrevilla@cubiqfoods.com (R.R.)

* Correspondence: analia.bortolozzi@iibb.csic.es

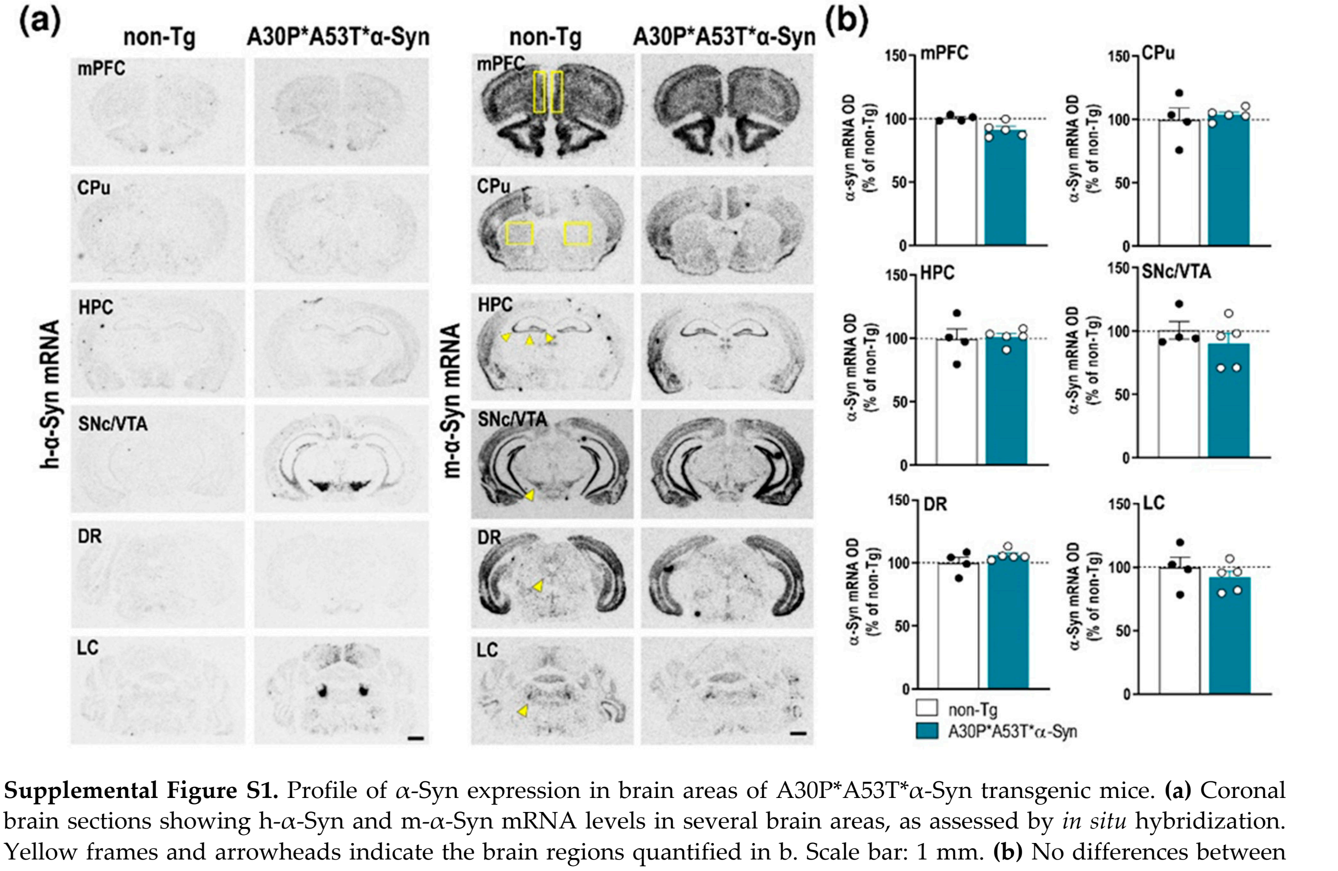


**Supplemental Figure S1.** Profile of *α*-Syn expression in brain areas of A30P*A53T**α*-Syn transgenic mice. **(a)** Coronal brain sections showing h-*α*-Syn and m-*α*-Syn mRNA levels in several brain areas, as assessed by *in situ* hybridization. Yellow frames and arrowheads indicate the brain regions quantified in b. Scale bar: 1 mm. **(b)** No differences between groups were detected for m-*α*-Syn RNA expression in all brain areas analyzed. Data are represented as the mean ± SEM. Abbreviations: mPFC, medial prefrontal cortex; CPu, caudate putamen; HPC, hippocampus; SNc, substantia nigra compacta; VTA, ventral tegmental area; DR, dorsal raphe nucleus; LC, locus coeruleus.

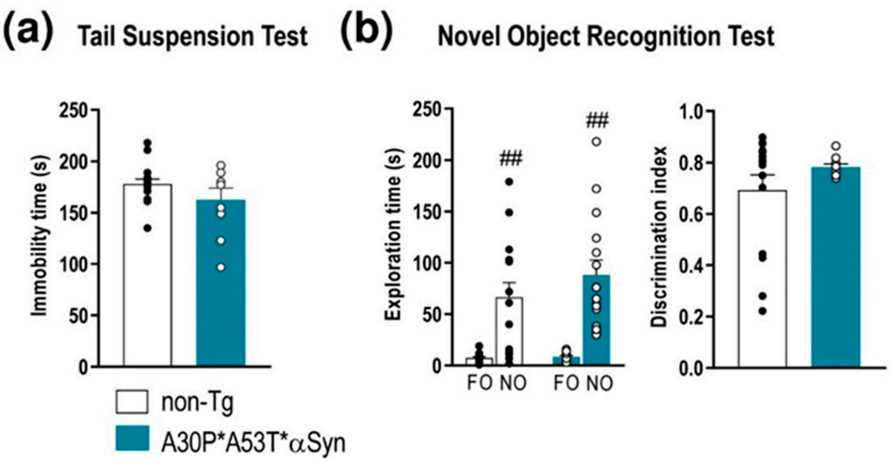


**Supplemental Figure S2. (a)** Both A30P*A53P*α-Syn and non-Tg mice showed a comparable immobility time in the tail suspension test suggesting a non-depressed phenotype. **(b)** A30P*A53P*α-Syn transgenic mice showed similar times of exploration between familiar and novel objects (FO and NO, respectively) compared to non-Tg mice. Data are expressed as the mean ± SEM, n = 10-15 mice/group. Two-way ANOVA and Sidak's multiple comparisons test, ## p < 0.01 compared to FO.

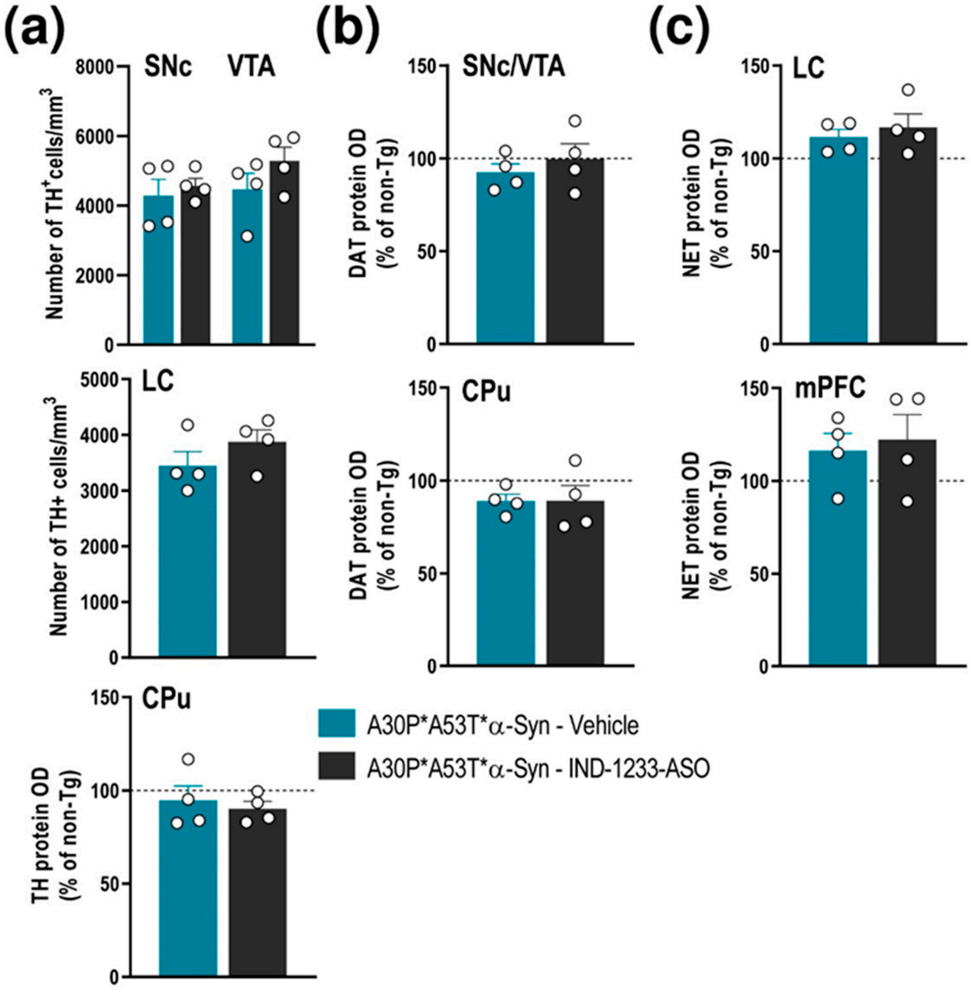


**Supplemental Figure S3.** Absence of the DA and NE neurotoxicity after intracerebroventricular administration of IND-1233-ASO. **(a)** Bar graphs show no differences in the number of SNc and VTA TH$^+$ cells or density of TH$^+$ striatal terminals. **(b,c)** Bar graphs show no differences in the DAT (b) and NET (c) protein density in DA and NE neuronal bodies and their brain projection areas. Values are the mean ± SEM, n=4 mice/group.

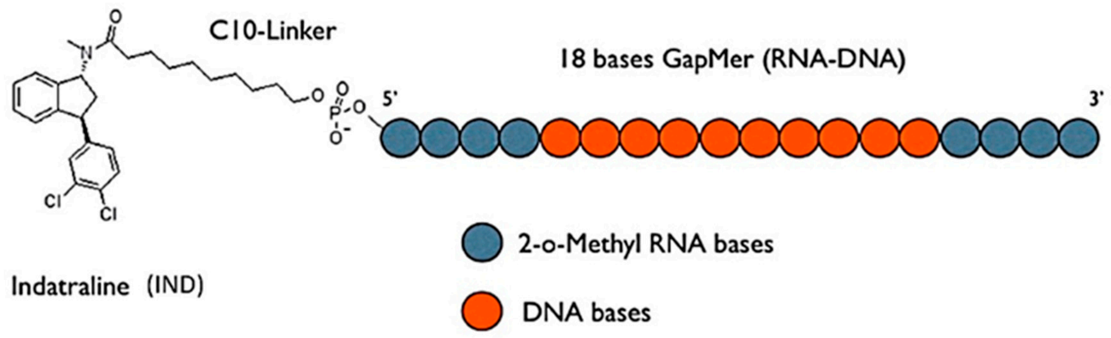


**Supplemental Figure S4.** Schematic representation of IND-1233-ASO molecule. The ASO consists of an antisense GapMer of 18-mer single stranded DNA molecule with four 2′-O-methyl RNA bases at both ends to protect the internal DNA from nuclease degradation and improve the binding to the target mRNA. Indatraline hydrochloride (major inhibitor of monoamine transporters) was conjugated to 5′-carboxy-C10 modified oligonucleotide through an amide bond.
